# *OutbreakTools*: A new platform for disease outbreak analysis using the R software

**DOI:** 10.1016/j.epidem.2014.04.003

**Published:** 2014-06

**Authors:** Thibaut Jombart, David M. Aanensen, Marc Baguelin, Paul Birrell, Simon Cauchemez, Anton Camacho, Caroline Colijn, Caitlin Collins, Anne Cori, Xavier Didelot, Christophe Fraser, Simon Frost, Niel Hens, Joseph Hugues, Michael Höhle, Lulla Opatowski, Andrew Rambaut, Oliver Ratmann, Samuel Soubeyrand, Marc A. Suchard, Jacco Wallinga, Rolf Ypma, Neil Ferguson

**Affiliations:** a MRC Centre for Outbreak Analysis and Modelling, Department of Infectious Disease Epidemiology, School of Public Health, Imperial College London, United Kingdom; b Immunisation, Hepatitis and Blood Safety Department, Public Health England, London, United Kingdom; c MRC Biostatistics Unit, Institute of Public Health, University Forvie Site, Cambridge, United Kingdom; d Mathematical Modelling of Infectious Diseases Unit, Institut Pasteur, Paris, France; e Centre for the Mathematical Modelling of Infectious Diseases, Department of Infectious Disease Epidemiology, London School of Hygiene and Tropical Medicine, United Kingdom; f Department of Mathematics, Imperial College London, United Kingdom; g Department of Veterinary Medicine, University of Cambridge, United Kingdom; h Interuniversity Institute of Biostatistics and Statistical Bioinformatics, Hasselt University, Hasselt, Belgium; i Centre for Health Economic Research and Modelling Infectious Diseases, Vaccine and Infectious Disease Institute, University of Antwerp, Antwerp, Belgium; j MRC - University of Glasgow Centre for Virus Research, Institute of Infection, Inflammation and Immunity, College of Medical, Veterinary and Life Sciences, University of Glasgow, Glasgow, United Kingdom; k Department of Mathematics, Stockholm University, Stockholm, Sweden; l Pharmacoepidemiology and Infectious Diseases Unit, Université de Versailles Saint Quentin EA4499/Institut Pasteur, Paris, France; m Institute of Evolutionary Biology, Center for Immunity, Infection and Evolution, University of Edinburgh, United Kingdom; n INRA, UR546 Biostatistics and Spatial Processes, Avignon 84914, France; o Departments of Biomathematics and Human Genetics, David Geffen School of Medicine at UCLA, University of California, Los Angeles, CA, USA; p Department of Biostatistics, UCLA Fielding School of Public Health, University of California, Los Angeles, CA, USA; q Center for Infectious Disease Control, National Institute of Public Health and the Environment, Bilthoven, The Netherlands; r Department of Infectious Disease Epidemiology, School of Public Health, Imperial College London, United Kingdom; s Wellcome Trust Sanger Institute, Wellcome Trust Genome Campus, Hinxton, Cambridge, United Kingdom

**Keywords:** Software, Free, Bioinformatics, Epidemiology, R, Epidemics, Public health, Infectious disease

## Abstract

The investigation of infectious disease outbreaks relies on the analysis of increasingly complex and diverse data, which offer new prospects for gaining insights into disease transmission processes and informing public health policies. However, the potential of such data can only be harnessed using a number of different, complementary approaches and tools, and a unified platform for the analysis of disease outbreaks is still lacking. In this paper, we present the new R package *OutbreakTools*, which aims to provide a basis for outbreak data management and analysis in R. *OutbreakTools* is developed by a community of epidemiologists, statisticians, modellers and bioinformaticians, and implements classes and methods for storing, handling and visualizing outbreak data. It includes real and simulated outbreak datasets. Together with a number of tools for infectious disease epidemiology recently made available in R, *OutbreakTools* contributes to the emergence of a new, free and open-source platform for the analysis of disease outbreaks.

## Introduction

Infectious disease outbreak investigation is a complex task in which a variety of data sources can be exploited for attempting to uncover the spatio-temporal dynamics and transmission pathways of a pathogen in a population. These data can include information on cases’ symptoms, their contacts, results of diagnostic tests and, increasingly, pathogen genetic sequences. Such rich and diverse data offer unprecedented prospects for understanding the process of disease transmission and ultimately designing adapted containment strategies and prophylaxis.

Dedicated methodological approaches are traditionally used to analyze different types of data separately, and can exploit information such as the generation time distribution and the timing of symptom onsets (Wallinga and Teunis, 2004; Hens et al., 2012), contact patterns amongst individuals (Calatayud et al., 2010; Cauchemez et al., 2011), geographic locations of the cases (Truscott et al., 2007; Chis Ster and Ferguson, 2007), or pathogen genetic sequences (Vega et al., 2004; Jombart et al., 2011; Harris et al., 2013). Interestingly, the advent of genetic data has also triggered a number of methodological developments aiming to exploit different types of data simultaneously (Ypma et al., 2012; Morelli et al., 2012; Teunis et al., 2013; Jombart et al., 2014; Mollentze et al., 2014). Unfortunately, few of these approaches are widely available to the community as computer software, and a unified platform for the analysis of disease outbreaks is still lacking.

Because it is free, open-source, and hosts the largest collection of tools for statistical analysis, the R software environment (R Core Team, 2013a) appears an ideal host for the development of such a platform. Besides dedicated packages for e.g. advanced linear modelling (Faraway, 2004), time series (Cowpertwait and Metcalfe, 2009), spatial processes (Bivand et al., 2008), multivariate methods (Karatzoglou et al., 2004; Zou and Hastie, 2012; Dray and Dufour, 2007), genetic data analysis (Paradis et al., 2004; Jombart, 2008; Jombart and Ahmed, 2011; Paradis, 2010) and graphics (Wickham, 2009), R offers the full flexibility of an interpreted computer language, allied with the possibility of calling upon precompiled routines, e.g. in C, C++ or Fortran, whenever computationally intensive tasks need to be undertaken. R is already hosting a growing number of packages for infectious disease epidemiology, including *surveillance* (Höhle, 2007) for temporal and spatio-temporal modelling (including outbreak detection), *R0* (Obadia et al., 2012), *TreePar* (Stadler and Bonhoeffer, 2013) and *EpiEstim* (Cori et al., 2013) for reproduction number estimation, and *outbreaker* (Jombart et al., 2014) for transmission tree reconstruction.

To ensure coherence between these different approaches and promote further developments, basic tools for storing and handling outbreak data are needed. In order to fill this gap, a community of epidemiologists, modellers, statisticians and bioinformaticians has developed the R package *OutbreakTools*. Here, “outbreak data” is defined as the above-described collection of data originating from a set of outbreak cases. This software, initiated during a hackathon for the analysis of disease outbreaks in R (http://sites.google.com/site/hackoutwiki/), provides object classes implementing a flexible and coherent representation of outbreak data, alongside procedures to manipulate, summarize and visualize these data. In this paper, we provide an overview of the main features of *OutbreakTools*, and discuss the future of R as a platform for the analysis of outbreak data.

## Results

The main purpose of *OutbreakTools* is to provide a coherent yet flexible way of storing outbreak data. To achieve this goal, a new formal (S4) class ‘*obkData*’ (short for ‘outbreak data’) has been developed. This class uses different slots (Table 1) to store individual meta data (e.g. age, sex), time-stamped observations made on the individuals (e.g. fever, swab results, or answers on food exposures from questionnaires), contacts between patients, DNA sequences of the pathogen, phylogenetic trees, and contextual data at the population level (e.g. school closures, climatic variables). Complex data structures such as dynamic contact networks or DNA sequences from different genes are respectively stored using the new classes ‘*obkContacts*’ and ‘*obkSequences*’.

To promote interoperability, *okbData* objects can be created from standard input files via procedures already available in R. Data tables can be imported from text files (extensions ‘.txt’ and ‘.csv’), from other statistical software using the package *foreign* (R Core Team, 2013b), or from XML files using the package XML (Butts, 2008). Aligned DNA sequences in FASTA format can be read using *ape* (Paradis et al., 2004) or *adegenet* (Jombart, 2008; Jombart and Ahmed, 2011), and phylogenetic trees can be imported from Newick or NEXUS format using *ape* (Paradis et al., 2004). To ensure that *obkData* objects are readily compatible with other R packages, existing classes have been used for storing data whenever possible: the class *‘DNAbin*’ for DNA sequences (Paradis et al., 2004), the classes ‘*network*’ and ‘*networkDynamic*’ for contact data (Butts, 2008), and the class ‘*phylo*’ for phylogenetic trees (Paradis et al., 2004).

Considerable efforts have been made to ensure that these different pieces of information are stored in a coherent way. The use of a formal (S4) class system offers multiple advantages in this respect, as it allows one to accurately define the object's content, and to perform consistency checks between the different data sources when the object is created. This means, for instance, that individuals documented in the contact or symptom data will be linked, through unique individual identifiers, to available individual meta-data, or that tips of the trees will be linked to existing DNA sequences whenever possible. Similarly, dates provided in different formats are automatically standardized, and sequences of the same genes are checked for consistent length. As *obkData* objects allow for coherent data storage and can be saved easily as compressed R objects (using the function save), they also offer a new and efficient way of sharing data amongst collaborators and making studies reproducible after publication.

Despite this complex data structure, accessing information stored in *obkData* objects is facilitated by a large number of accessors. These functions allow for the retrieval of specific data (get.data), including sampling dates (get.dates), contacts (get.contacts), individual meta-data (get.individuals) or DNA sequences from given genes (get.dna), without requiring knowledge about the internal data structure. Importantly, decoupling the access to information from the internal data storage also ensures long-term code portability: future changes in the data structure will not affect results as long as accessors return the same information. This approach will enable future developments of the *obkData* class and allow for the incorporation of new types of data. Besides accessors, data handling is also facilitated by a subsetting procedure (function subset) which allows one to isolate data for given sets of individuals, samples, genes, sequences, or from a given time window.

**Fig. 1:**
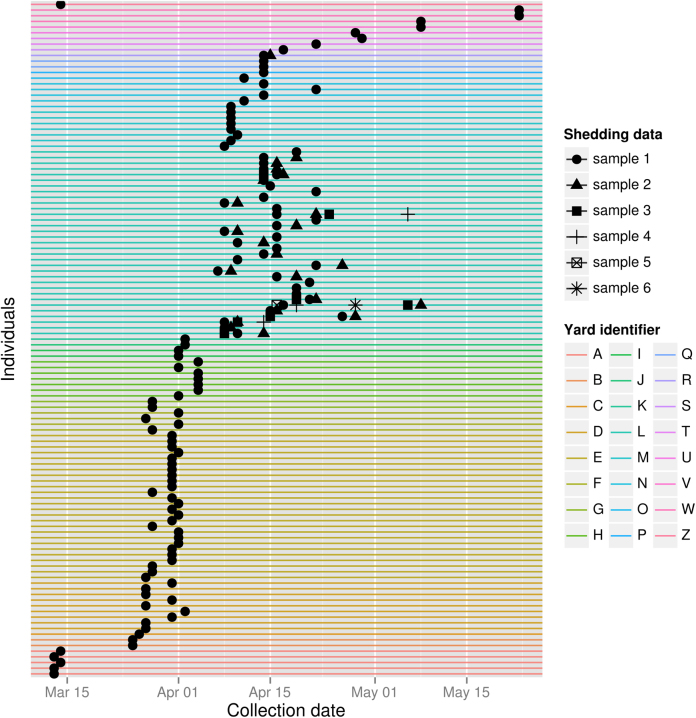
Timeline of samples of the Newmarket equine influenza outbreak (HorseFlu dataset). This figure represents the temporal distribution of the VIRAL shedding samples gathered during the outbreak. Each horizontal line represents an individual. Individuals are sorted and coloured by yard. Repeated samples gathered on the same individual are represented using different symbols. The code for reproducing this figure is provided in Appendix 1.

**Fig. 2:**
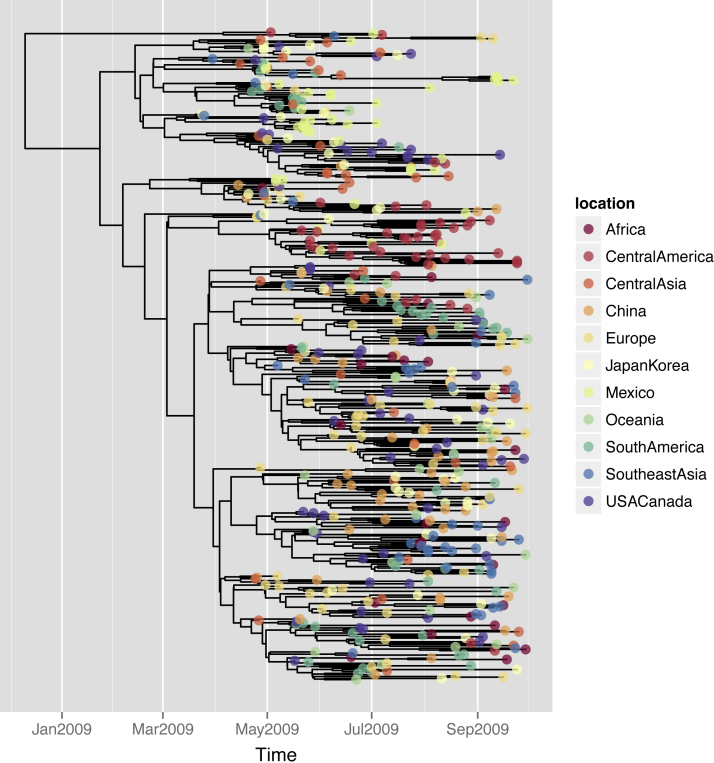
Phylogeny of pandemic influenza H1N1 sequences (FluH1N1pdm2009 dataset). This phylogenetic tree based on 514 hemagglutinin segments of pandemic influenza H1N1 was plotted using the function plotggphy. The code for reproducing this figure is provided in Appendix 1.

The information contained in *obkData* objects can be easily visualized using options of the generic function plot, or directly using dedicated functions. Individual timelines can be used to visualize course of illness and collection dates of samples for each individual (function plotIndividualTimeline, Fig. 1), maps can be drawn to assess the geographic distribution of the cases (function plotGeo), contact data can be visualized as graphs (function plotfor *obkContacts* objects), and genetic data can be visualized as phylogenies (function plotggphy, Fig. 2) and minimum spanning trees (function plotggMST). Most of these graphs take advantage of the high-quality customisable graphics implemented in ggplot2 (Wickham, 2009).

While *OutbreakTools* focuses on storing, handling and visualizing data, the package also implements basic tools for data analysis. Adapted summaries (function summary) have been implemented to provide quick insights into the data, make.phylocan be used to obtain phylogenies for all genes of the dataset, and get.incidence can be used to compute incidence from dates of symptom onsets, but also from any time-stamped data. In the latter situation, positive cases can be defined from either quantitative or categorical data, by specifying a range of numerical values, a list of character strings or even regular expressions. In practice, this allows for the computation of incidence based on any symptom data or sample analysis. This feature therefore allows for a direct use of procedures implemented in *R0* (Obadia et al., 2012) or *EpiEstim* (Cori et al., 2013) for estimating reproduction numbers.

**Fig. 3:**
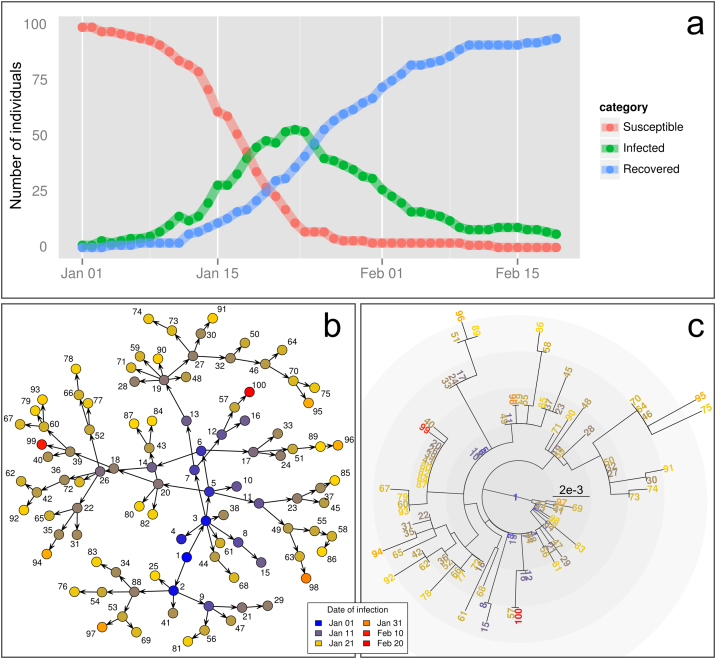
Simulated outbreak using simuEpi. This outbreak was simulated under a SIR model with 100 hosts, contact rate *β* = 0.005 and recovery rate *ν* = 0.1. (a) Dynamics of the outbreak showing the numbers of susceptibles, infected and recovered over time. (b) Transmission tree, where each dot is a labelled case with colours representing the date of infection. (c) Neighbour-Joining phylogeny reconstructed from the simulated DNA sequences, ladderized and rooted to the first case. The code for reproducing these figures is provided in Appendix 1.

To illustrate its features, *OutbreakTools* is released with both simulated and empirical datasets, including 514 annotated DNA sequences of the 2009 influenza pandemic (dataset FluH1N1pdm2009, Fig. 2) and data from a large Newmarket (UK) outbreak of equine influenza (dataset HorseFlu; Hughes et al., 2012, Fig. 1). Finally, *OutbreakTools* also includes a simulation tool (function simuEpi) which allows for the generation of outbreaks (including pathogen genome sequences) under a standard SIR model (Fig. 3), and can easily be extended to use other models (e.g. SIS, SEIR). *OutbreakTools* is documented in a 50-page manual and released with a tutorial introducing the data structures and the main functionalities of the package.

## Discussion

While a number of packages for infectious disease epidemiology have recently been developed in the R software (Jombart et al., 2014; Obadia et al., 2012; Stadler and Bonhoeffer, 2013; Cori et al., 2013), basic tools for storing, handling and visualizing outbreak data have so far been lacking. *OutbreakTools* fills this gap by implementing new formal classes allowing for a coherent yet flexible representation of disease outbreak data, alongside a number of functions for manipulating and visualizing that data. As such, it represents a significant step towards building a comprehensive platform for outbreak analysis in R. The collaborative and open nature of this project, together with the possibility of modifying internal data structures seamlessly for the user, ensures that *OutbreakTools* will be able to evolve and adapt to incorporate new types of data and approaches used for outbreak analysis.

The new availability of basic tools for outbreak analysis will hopefully encourage the further development of tools for investigating epidemics. It should in particular facilitate the implementation of novel integrative approaches able to exploit various types of data simultaneously (Ypma et al., 2012; Morelli et al., 2012; Teunis et al., 2013; Mollentze et al., 2014). Comparing the tools emerging from this still-burgeoning methodological field will likely be useful, as was recently demonstrated by the HIV modelling community (Eaton et al., 2012). In this respect, the existence of a unified platform for the analysis of disease outbreaks should provide the common ground needed for such comparisons to be drawn. More generally, the provision of a coherent structure for storing outbreak data will drastically improve the ease of data exchange amongst collaborators and hopefully encourage data sharing within the community.

Arguably, the choice of R for developing a new platform for outbreak analysis may initially appeal mostly to a community of R experts, and considerable efforts should be made to reach as broad an audience as possible. First, providing free tutorials and teaching material is paramount for making new tools accessible to the community at large. This is the objective of the “*R-epi project*” (http://sites.google.com/site/therepiproject/), a website developed collaboratively and aiming to provide free resources for the analysis of disease outbreaks primarily in R, but also using other free software. Interestingly, recent developments such as the package *shiny* (Beeley, 2013) dramatically aid in the development of user-friendly web interfaces running R tools. Such approaches could be considered for reaching out to an even broader audience and trying and maximize the availability of leading-edge methods for epidemics analysis to the community at large, including not only modellers and statisticians, but also epidemiologists and public health agencies.

## Resources

**Availability:** *OutbreakTools* 0.1–0 is distributed on CRAN (http://cran.r-project.org/) and available for R 3.0.2 on Windows, Mac OSX, and Linux platforms. It can be installed as any other package using the graphical user interface or typing the instruction: install.packages(“OutbreakTools”)

**Licence:** GNU General Public Licence (GPL) ≥2.

**Website:** http://sites.google.com/site/therepiproject/r-pac/about

**Documentation:** besides the usual package documentation, *OutbreakTools* is released with a tutorial which can be opened by typing: vignette(“OutbreakTools”). More documentation can be found on the project's website.

**Development:** the development of *OutbreakTools* is hosted on Sourceforge: http://sourceforge.net/projects/hackout/

New contributions are welcome and encouraged.

## Figures and Tables

**Figure:**
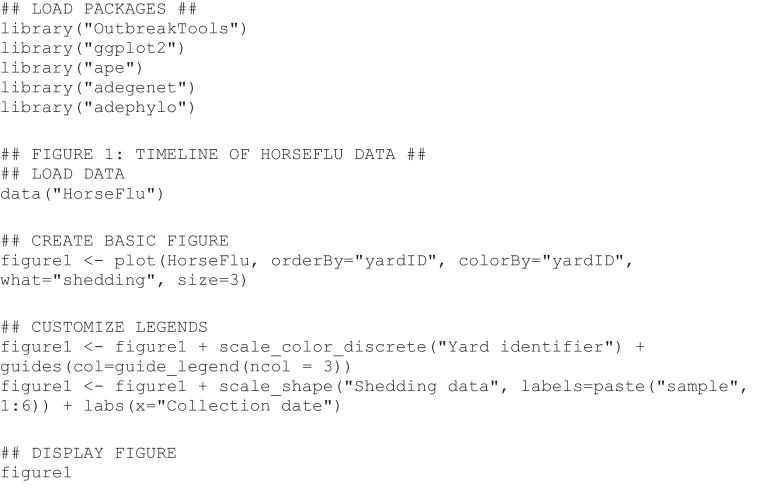


**Figure:**
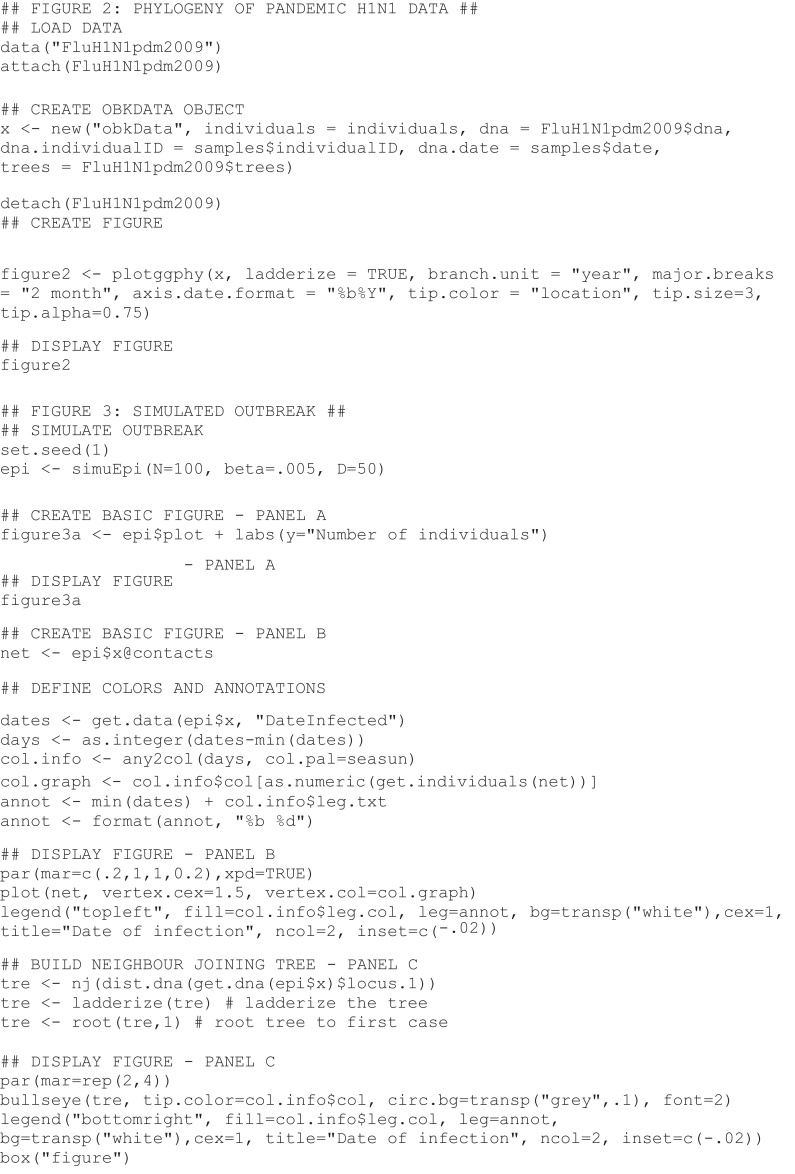


**Table 1:** Content of the formal (S4) class ‘*obkData*’. Instances of the class *obkData* can store a variety of data in the indicated slots. Filling the slots is optional, and empty slots are all NULL.

Slot name	Content
@individuals	*data.frame* containing patient meta-data (e.g. age, sex).
@records	*list* of *data.frame* containing time-stamped observations made on cases (e.g. fever, swab results); allows for repeated observations on the same individual.
@dna	*obkSequences* object containing pathogen genetic sequences for one or several genes with recorded collection dates; uses the class ‘*DNAbin’* to store sequences; allows for multiple sequences for the same cases.
@contacts	*obkContacts* object storing contact data between patients, stored as a static or dynamic network; uses the classes ‘*network’* and ‘*networkDynamic*’.
@trees	*multiphylo* object storing one or several phylogenetic trees of pathogen genomes; uses the class ‘*phylo*’ to store trees.
@context	a *list* of *data.frames* contextual data relevant at a population level (e.g. school closure)
